# Inhibitory Effects of Ginsenoside Ro on the Growth of B16F10 Melanoma via Its Metabolites

**DOI:** 10.3390/molecules24162985

**Published:** 2019-08-17

**Authors:** Si-wen Zheng, Sheng-yuan Xiao, Jia Wang, Wei Hou, Ying-ping Wang

**Affiliations:** 1 Institute of Special Animal and Plant Sciences, Chinese Academy of Agricultural Sciences, Changchun 130112, China; 2 National & Local Joint Engineering Research Center for Ginseng Breeding and Development, Changchun 130118, China; 3 School of Pharmaceutical Sciences Changchun University of Chinese Medicine, Changchun 130117, China

**Keywords:** ginsenoside Ro, metabolites, anti-tumour, B16F10

## Abstract

Ginsenoside Ro (Ro), a major saponin derived and isolated from *Panax ginseng* C.A. Meyer, exerts multiple biological activities. However, the anti-tumour efficacy of Ro remains unclear because of its poor in vitro effects. In this study, we confirmed that Ro has no anti-tumour activity in vitro. We explored the anti-tumour activity of Ro in vivo in B16F10 tumour-bearing mice. The results revealed that Ro considerably suppressed tumour growth with no significant side effects on immune organs and body weight. Zingibroside R1, chikusetsusaponin IVa, and calenduloside E, three metabolites of Ro, were detected in the plasma of Ro-treated tumour-bearing mice and showed excellent anti-tumour effects as well as anti-angiogenic activity. The results suggest that the metabolites play important roles in the anti-tumour efficacy of Ro in vivo. Additionally, the haemolysis test demonstrated that Ro has good biocompatibility. Taken together, the findings of this study demonstrate that Ro markedly suppresses the tumour growth of B16F10-transplanted tumours in vivo, and its anti-tumour effects are based on the biological activity of its metabolites. The anti-tumour efficacy of these metabolites is due, at least in part, to its anti-angiogenic activity.

## 1. Introduction

Skin cancer is a common type of cancer with an increasing incidence rate and public health burden [1]. As the most aggressive form of skin cancer, melanoma is a devastating disease as it is highly metastatic and has a poor prognosis once metastasis has occurred; there is no therapy that has consistently improved overall survival in patients with metastatic melanoma [2,3]. Melanoma originates from melanocytes, specialised pigmented cells found in the epidermis [4]. Patients with this disease have an increased risk of developing subsequent primary melanoma [5]. Its incidence has increased faster than that of any other cancer type during recent decades. In 2018, 287,723 new cases were diagnosed, with 60,712 associated deaths around the world [6]. National Cancer Institute’s Surveillance, Epidemiology, and End Results (SEER) is an authoritative source for cancer statistics in the United States. According to the SEER database, malignant melanoma is the fifth most common cancer for both men and women, with a rising incidence and an estimated 96,480 new cases in 2019 in the United States alone [7]. Therefore, development of potential treatments for melanoma is an important clinical problem.

Chemotherapy is one of the most effective approaches to cure malignant melanoma, but its toxicity and the development of drug resistance represents important limits in determining its efficacy [8]. Enucleation can significantly worsen a melanoma patient’s quality of life, considering their poor prognosis and high fees [9]. In addition, indications of radiotherapy are currently reduced, since melanoma is traditionally considered to be a radioresistant tumour [10]. Therefore, the development of novel agents and strategies for malignant melanoma has become a priority and an important global medical issue. Accordingly, there is a need to identify alternative approaches that can improve survival and quality of life.

Natural compounds derived from potential herbs were able to effectively interfere with tumour progression, inhibit angiogenesis, and block metastasis [11]. Due to its affordability and good therapeutic efficacy, natural products have garnered increasing attention as cancer treatments [12,13]. Natural products could be used as adjuvants to existing treatments to improve efficacy and/or reduce drug-induced toxicity [14]. These compounds can be clinically used on their own or in combination with other natural products or drugs [15]. The therapeutic potential in melanoma growth and metastasis has been demonstrated for several species of natural compounds including alkaloids, polyphenols, vitamin-related compounds, terpenoids, peptides, and herbal extracts [16].

Ginseng (*Panax ginseng* C.A. Meyer) is traditionally used as an herbal medicine in Far Eastern countries and has gained popularity in Western countries in recent decades [17]. It is also frequently used as tonics and dietary supplements [18]. Ginsenosides are the main bioactive ingredients responsible for various pharmacological effects [19,20] and can be divided into three types: protopanaxadiol (PPD), protopanaxatriol (PPT), and oleanolic acid (OA) [21]. During the past 30 years, great efforts have been made to identify ginsenosides with anti-cancer activity, especially PPD- and PPT-type ginsenosides. These compounds possess anti-cancer activity in multiple cell types including hepatic [22,23], pulmonary [24,25], prostate [26,27], colonic [28], ovarian [29,30], gastric [31,32], glioma [33,34], leukaemia [35,36], and breast [37,38] cancer cells. In particular, PPD-type ginsenosides have higher anti-cancer activity than PPT-type [39,40,41]. Although the anti-tumour activities of PPD- and PPT-type ginsenosides have been relatively well documented, OA-type ginsenosides have been less studied.

Ginsenoside Ro (Ro) is a highly abundant OA-type ginsenoside in ginseng [42], which sensitises esophageal cancer cells to 5-fluorouracil-induced cell death by inhibiting autophagosome–lysosome fusion [43]. It also possesses a potential skin anti-photoaging property against ultraviolet B radiation in fibroblasts [44]. Moreover, Ro has anti-complementary [45], anti-inflammatory [46,47], anti-hepatitic [48], anti-diabetogenic [49], and hair re-growth promoting [50] activities. OA is an aglycone of Ro, in fact, OA and its derivatives have proven effective for inducing apoptosis and inhibiting the proliferation of cancer cells [51,52,53,54,55,56]. The cytotoxicity of OA has been established in several cell lines, including the A549, SK-OV-3, SK-MEL-3, HCT15, HONE-1, KB, and HT29 cell lines, with IC50s ranging from 12.1 to 18.5 μg/mL [57,58]. Furthermore, OA is also effective in inhibiting the invasion and metastasis of tumour cells; OA decreased the expression of angiogenic vascular endothelial growth factor (VEGF) and decreased the development of melanoma-induced lung metastasis [59,60]. The anti-tumour effect of OA is evident, so how about Ro? Due to the fact that Ro has not shown significant cytotoxicity in tumour cells in vitro [61], there have been few attempts to evaluate the anti-tumour activity in vivo.

Here, we determined if Ro has efficacy against tumours in mice. To this end, B16F10 melanoma cells were subcutaneously injected into experimental mice, after which Ro was intraperitoneally administered to the tumour-bearing mice for 15 days. The metabolites of Ro were analysed using high-performance liquid chromatography-mass spectrometry (HPLC-MS). Subsequently, the efficacy of Ro and its metabolites was investigated in vivo and in vitro. The results showed that Ro has anti-tumour activity, and its efficacy is through its metabolites. The anti-tumour efficacy of these metabolites is due, at least in part, to its anti-angiogenic activity. The findings in the present study provide a theoretical basis for Ro to be used in the clinic for melanoma treatment and indicate that Ro has the potential to be an efficient anticancer drug in the treatment of melanoma in the future.

## 2. Results

### 2.1. Anti-Tumour Effects of Ro In Vivo

**Figure 1:**
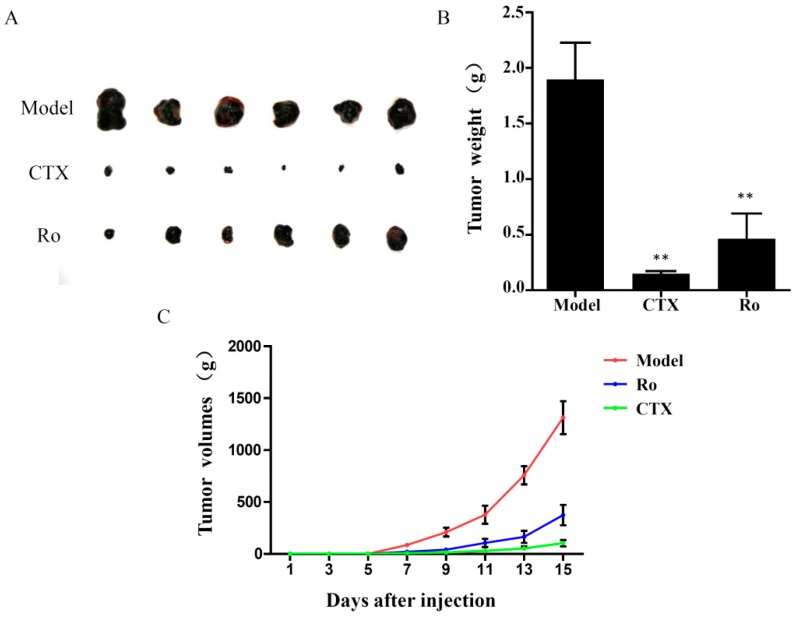
Effects of Ro on tumour growth in B16F10 tumour-bearing mice. (**A**) Image of tumour masses. (**B**) Weight of tumour masses. (**C**) Tumour volume changes in B16F10 tumour xenograft mice. ** *p* < 0.01 vs. model group. CTX: cyclophosphamide.

The anti-tumour effects of Ro were evaluated in a xenograft mouse model. Intraperitoneal injection of Ro (25 mg/kg) for 15 days resulted in a significant decrease in tumour weight compared to the model group (Figure 1). This finding revealed that 25 mg/kg Ro injection significantly reduced the tumour weight with a tumour inhibition rate of 75.7%. These results suggest that Ro considerably inhibits tumour growth in the xenograft mouse model. To determine if Ro administration resulted in any side effects on the immune system, we determined the thymus and spleen indices of the host animals at the end of the study. The results showed great weight loss of the thymus and spleen after cyclophosphamide (CTX, 20 mg/kg) treatment, which accounted for the immunosuppressive side effects caused by CTX during therapy (Table 1). However, the thymus and spleen indices in the Ro group were comparable to those in the normal group. Additionally, no significant loss of body weight was observed among the animals after treatment with Ro. These data suggested that Ro has no side effects on the immune organs and body weight in B16F10 tumour-bearing mice.

### 2.2. Effects of Ro on the Viability of B16F10 Melanoma Cells In Vitro

**Figure 2:**
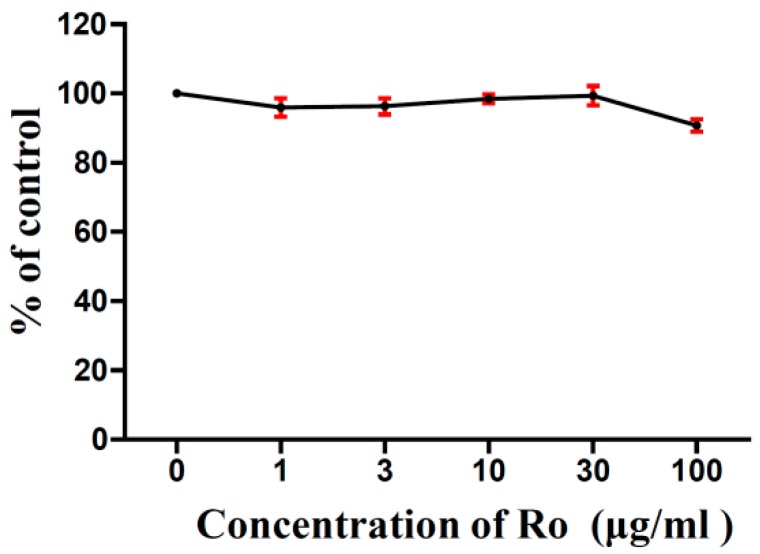
Effects of Ro on B16F10 melanoma cell viability. The results of the control (0 µg/mL) were normalised to 100%, and the results from Ro-treated cells were expressed as % of the control.

The effects of Ro on B16F10 cell viability were evaluated using the 3-(4,5-dimethylthiazol-2-yl)-2, 5-diphenyltetrazolium bromide (MTT) assay. B16F10 cells were exposed to various concentrations of Ro (0, 1, 3, 10, 30, and 100 μg/mL) for 24 h. The results are shown in Figure 2, and the MTT cell viability assay shows that Ro has little effect on B16F10 cell growth in vitro.

### 2.3. Determination and Identification of Metabolites in the Plasma of Tumour-Bearing Mice Treated with Ro

**Figure 3:**
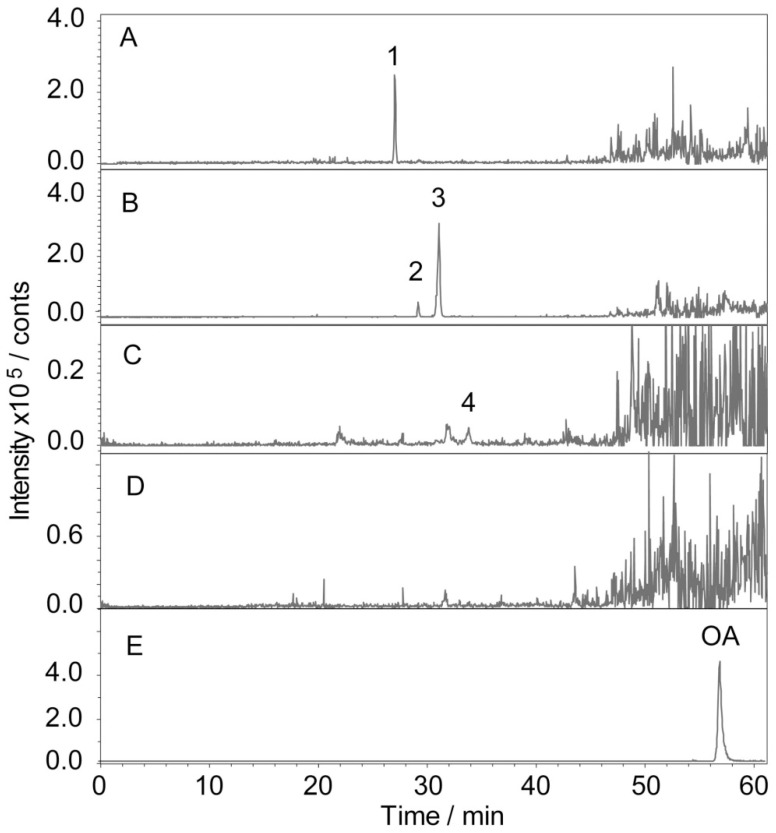
Representative chromatograms of Ro and its metabolites in the plasma. The tumour-bearing mice were administered Ro for 15 successive days, after which they were sacrificed, and the plasma was collected and evaluated. (**A**) Extracted ion chromatogram (EIC) at *m*/*z* 955.5; (**B**) EIC at *m*/*z* 794.5; (**C**) EIC at *m*/*z* 631.5; (**D**) EIC at *m*/*z* 455.5; (**E**) chromatogram of standard oleanolic acid (OA) (45.2 ng/inj, EIC at *m*/*z* 455.5). (1) Ro: ginsenoside Ro; (2) IVa: chikusetsusaponin IVa; (3) R1: zingibroside R1; (4) E: calenduloside E.

**Figure 4:**
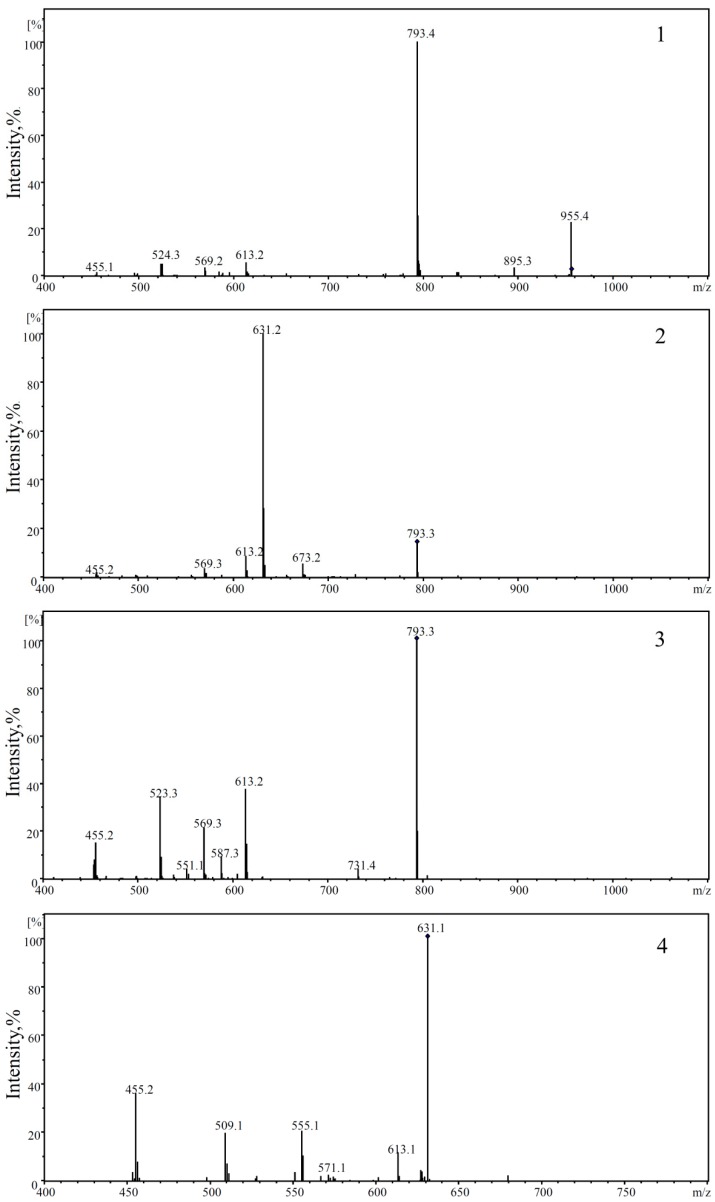
Negative collision-induced dissociation (CID) spectrum of compounds **1**–**4**. (1) Ro: ginsenoside Ro; (2) IVa: chikusetsusaponin IVa; (3) R1: zingibroside R1; (4) E: calenduloside E.

**Figure 5:**
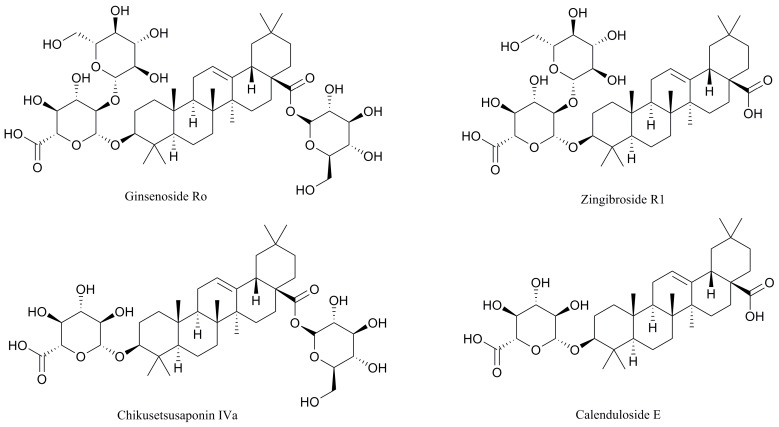
Structures of Ro, R1, IVa, and E.

After 15 consecutive days of Ro treatment, all mice were sacrificed, and the blood was collected. The plasma samples were prepared by centrifugation for 10 min at 3800 rpm and stored at −80 °C. Figure 3 shows the detection results of the plasma in tumour-bearing mice treated with Ro. The identification of the metabolites was based on retention times and MS/MS data. In the negative mode, the daughter ions of oleanolic glycosides were all observed from the cleavage of its glycosidic bond. A high abundance of fragments originated from cleavage of the glycolipid glycoside bond at position C-28. Conversely, a lower abundance of fragments originated from dissociation of the glucosyl ether bond. This result indicates that the glucosyl ester bond is more liable to be dissociated than the glucosyl ether. Therefore, fragments of deglycosylated can serve as a basis for determining the position of glycosylation [62]. The negative collision-induced dissociation (CID) spectrum of compounds **1**–**4** is shown in Figure 4. The retention time of compound **1** was 27.0 min (Figure 3A). It showed a deprotonated molecular ion at *m*/*z* 955 [M − H]^−^ in the negative all-scan MS spectrum, suggesting that its molecular weight was 956. The observation of deglycosylated fragments in the negative CID spectrum (shown in Figure 4-1) at *m/z* 793 [M − H − 162]^−^, *m*/*z* 613 [M − H − 342]^−^, and *m*/*z* 455 [M − H − 500]^−^ indicates that there are two hexoses and one hexuronic acid located on the aglycone. A high abundance of fragments was found at 793 [M − H − 162]^−^. Therefore, one molecule of hexose should be located at C-28. The fragment ion at *m*/*z* 455 [M − H − 500]^−^ was a characteristic fragment of OA. This observation suggests that the structure of compound 1 was Ro. Compounds **2** and **3** both showed a deprotonated molecular ion at *m*/*z* 793 [M − H]^−^, with retention times of 29.2 and 31.1 min, respectively. Thus, we inferred that they were isomers. The high abundance of a fragment in compound **2** was at 631 [M − H − 162]^−^, suggesting that it may be related to the location of the glycoside. The glucosyl ester bond was more liable dissociated than the glucosyl ether. Therefore, it was judged that one molecule of hexose was located at C-28 for compound **2**, and one molecule of hexanoic acid was attached to C-3. In contrast, the high abundance of the fragment in compound **3** was at 613 [M − H – 162 − 18]^−^, due to the glycosidic bond dissociating with a drop of water. Therefore, it was determined that the hexose and hexanoic acid contained in compound **3** were both located at the C-3 position. Both of them contained characteristic fragment ions *m*/*z* 455 [M − H − 338]^−^ of OA, which were identified as chikusetsusaponin IVa (IVa) and zingibroside R1 (R1) by the literature. Compound **4** showed a deprotonated molecular ion at *m*/*z* 631 [M − H]^−^ in the negative all-scan MS spectrum and also contained an OA characteristic fragment ion *m*/*z* at 455 [M − H − 176]^−^. Simultaneously, fragment ions *m*/*z* 613 [M − H − 18]^−^, *m*/*z* 555 [M − H − 76]^−^, and *m*/*z* 509 [M − H − 122]^−^ were produced by cleavage of the glycosidic ring, proving that the glycosidic bond was stable. The results indicate that the hexanoic acid is located at the C-3 position and is identified as calenduloside E (E) by the literature. The MS results show that the fragment ions of the abovementioned compounds are similar; thus, IVa, R1, and E are determined to be metabolites of Ro. R1 was found to be the most abundant metabolite in the plasma. The signal intensity of R1 was approximately two-fold that of Ro, 10-fold that of IVa, and 100-fold that of E. Glycosyl oleanolate and OA were not detected in the plasma (Figure 3), possibly due to interference from the high background signals. The structures of Ro and its metabolites are shown in Figure 5.

### 2.4. Effects of R1, IVa, and E on the Viability of B16F10 Melanoma Cells In Vitro

**Figure 6:**
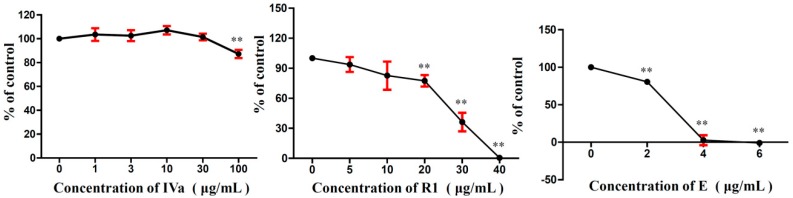
Effects of IVa, R1, and E on B16F10 melanoma cells in vitro. The results of the control (0 µg/mL) were normalised to 100%, and the results from treated cells were expressed as % of the control (* *p* < 0.05, ** *p* < 0.01).

The cytotoxic activities of metabolites of Ro are shown in Figure 6. The MTT assay showed that R1 and E induced dose-dependent cytotoxicity in B16F10 cells. The half maximal inhibitory concentration (IC50) of R1 and E were 24.52 and 2.58 µg/mL, respectively. IVa showed no cytotoxic effects at 1–30 µg/mL, and the viability was slightly decreased at a high concentration (100 µg/mL).

### 2.5. Anti-Tumour Effects of R1, IVa, and E In Vivo

**Figure 7:**
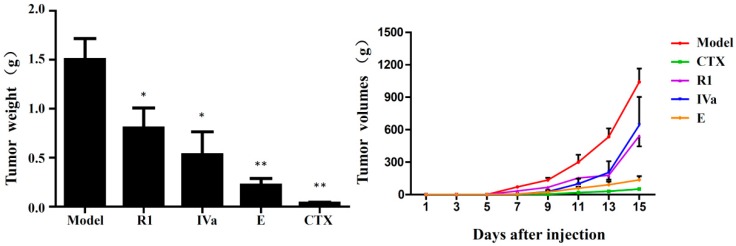
Effects of Ro metabolites on changes in tumour weight and tumour volume in B16F10 tumour xenograft mice. R1 (25 mg/kg), IVa (25 mg/kg), and E (5 mg/kg) were intraperitoneally injected into mice for 15 consecutive days. Values are expressed as the mean ± standard deviation (SD) of six mice.

The in vivo anti-tumour activities of R1, IVa, and E were evaluated in the same way as Ro. The anti-tumour effects of R1, IVa, and E on B16F10 tumour-bearing mice are summarised in Figure 7. At the end of the experiment, the average tumour weight in the model group was 1.50 g. Compared to the model group, the mean tumour weights in the R1 (25 mg/kg), IVa (25 mg/kg), and E (5 mg/kg) groups were significantly decreased to 0.80, 0.53, and 0.22 g, respectively. Accordingly, the tumour inhibitory rates of R1-, IVa-, and E-treated groups were 46.6%, 64.7%, and 85.4%, respectively. Figure 6 shows the tumour volume growth curves. The results indicate that the tumour volumes of mice in the model group rapidly increased during the 15 days of treatment, with the mean volumes reaching more than 1.04 × 10^3^ mm^3^ at the end of the experiment. In contrast, treatment with R1, IVa, E, and CTX significantly suppressed tumour growth (*p* < 0.05). From day 11, the average tumour volume in R1-, IVa-, E-, and CTX-treated mice increased relatively slowly.

### 2.6. Effects of R1, IVa, and E on Tube Formation of Human Umbilical Vein Endothelial Cells

**Figure 8:**
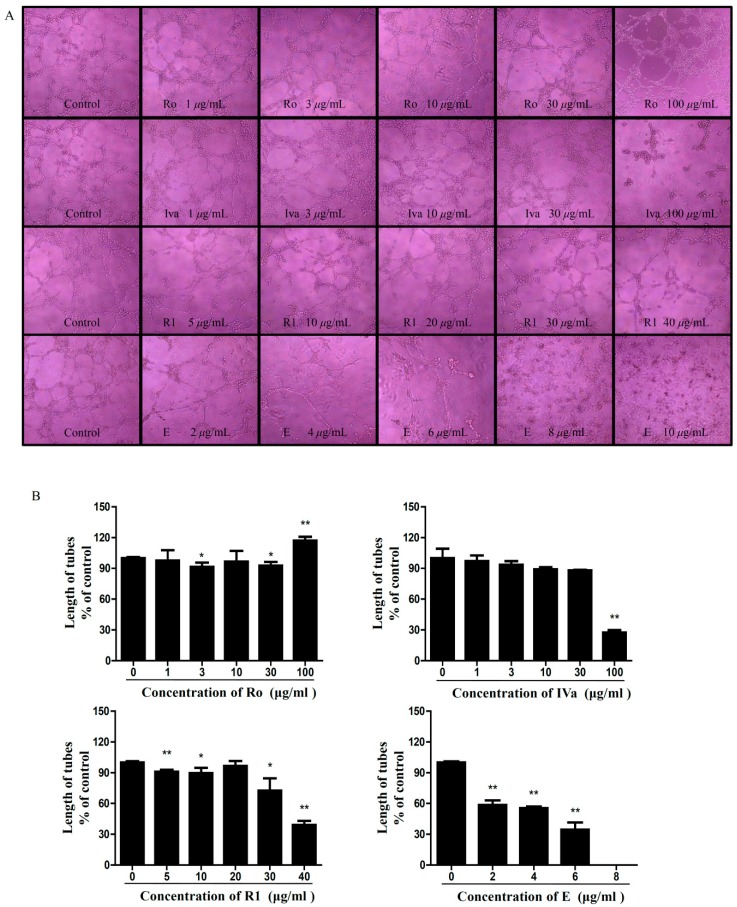
Results of tube formation. (**A**) The human umbilical vein endothelial cells (HUVECs) tubular structures were imaged under a microscope at 100× magnification. (**B**) Tube networks were quantified using Image-Pro Plus 6.0 software (Media Cybernetics, Silver Spring, MD, USA). The tube length was calculated (* *p* < 0.05, ** *p* < 0.01).

To examine the effects of Ro and its metabolites on crucial functions in blood vessel formation, we investigated whether the compounds decreased the formation of tubes by human umbilical vein endothelial cells (HUVECs) in vitro. Our results showed that IVa, R1, and E markedly inhibited HUVEC tube formation. Tube formation was imaged, and the tube length was determined (Figure 8). Low concentrations of Ro (1–30 μg/mL) had no effects on the tube formation of HUVECs. However, Ro stimulated tube formation at 100 μg/mL.

### 2.7. Effects of Ro in the Haemolysis Test

**Figure 9:**
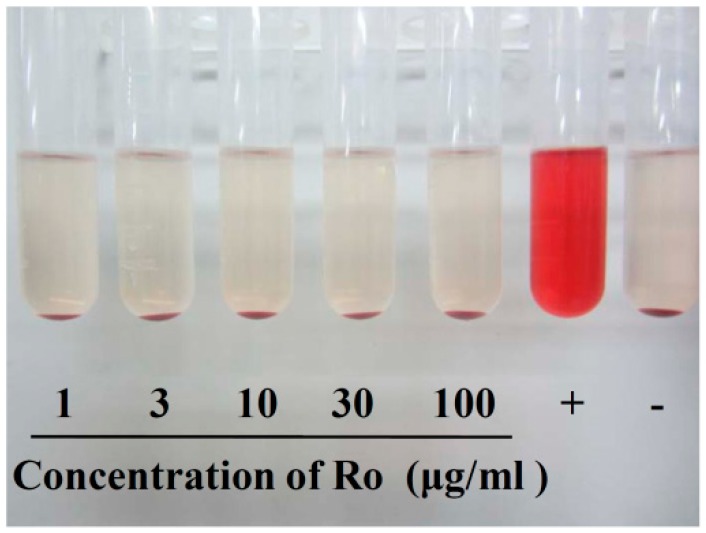
Haemolysis test result of Ro.

Haemolysis rate is an important parameter for evaluating blood compatibility, and less than 5% haemolysis is regarded as a nontoxic effect level [63]. Nearly no haemolysis was observed for the negative control (−), and complete haemolysis was observed for the positive control (+). The solution colour of Ro at different concentrations did not significantly change compared with the negative control group (Figure 9). It can be seen that the haemolysis ratio (HR%) was much lower than 5% (shown in Table 2), so we concluded that Ro exhibited good biocompatibility even at a high concentration of 100 μg/mL.

## 3. Discussion

Ginseng is a popular herbal medicine worldwide, and its anti-cancer activity has been proven by numerous studies. Asian ginseng (*Panax ginseng*) and American ginseng (*Panax quinquefolius*) are two species in ginseng genus [64]. Although most ginseng species contain both PPD- and PPT-type ginsenosides, their content vary greatly, leading to potentially different and unpredictable therapeutic effects [65]. In general, American ginseng contains higher level of PPD-type ginsenosides while Asian ginseng is more abundant in PPT-type ginsenosides. Thus, given the predominant PPD-type ginsenosides have higher anti-cancer activity than PPT-type, American ginseng may be more pharmacologically effective than Asian ginseng in terms of anticancer [66]. According to different processing methods, ginseng can be divided into fresh ginseng (FG), white ginseng (WG), red ginseng (RG), and black ginseng (BG) [67]. RG is obtained by steaming FG for a reasonable time, while WG is obtained by drying FG. BG is developed from WG by repetitive steaming nine times. Differences in pharmacological effects of the ginseng processed products can also be attributed to the heat-induced chemical degradation and conversion during the steaming treatment [67,68]. Steamed ginseng produces new constituents, not present in WG, such as (20(S)-, 20(R)-Rg3, Rk3, Rh4, Rk1, Rg5, etc.) [8,67]. After nine cycles of steaming, BG exhibits more powerful biological activities than WG or RG [69]; red ginseng has more potent anticancer effects than white ginseng [70].

It is worth mentioning that many bulky ginsenoside molecules are chemically transformed in the gut upon consumption and are absorbed as smaller metabolites [21]. These metabolites have more pharmacological activity than their parent compounds [71] and have high bioavailability. Taking PPD-type ginsenoside as an example, PPD-type ginsenoside is metabolized primarily to compound K (CK) by intestinal bacteria via the stepwise cleavage of sugar moieties [72]. Ginsenoside Rb1 (Rb1), one of the main components of ginseng, is a PPD-type ginsenoside consisting of a non-sugar component (aglycone) with a dammarane skeleton and a sugar component comprising of four sugar moieties [73]. When compared with Rb1, CK has significantly stronger anti-cancer effects [74]. However, previous studies have mainly investigated PPD- or PPT-type ginsenosides, OA-type ginsenosides have been less studied (the structures of Rb1, CK, Ro and its metabolites are summarized in Figure S1 and Table S1). Ro is a highly abundant ingredient in ginseng that acts as an OA-type ginsenoside. Due to its weak anti-tumour activity in vitro, Ro has received little attention by researchers. In this study, Ro had little effect on B16F10 cell viability. The result partially agreed with previous studies on the effects of ginsenosides on cell growth [61]. However, to our surprise, Ro showed high anti-tumour activity in vivo. The tumour volume and tumour weight of tumour-bearing mice were reduced after intraperitoneal injection of Ro.

In a previous study, Wang et al. [62] identified 14 metabolites of Ro in both the faeces and urine of rats after oral administration. Therefore, HPLC detection was done to identify the metabolites. Although OA was not detected in the plasma of the mice, it was detected in the urine of the rats after oral administration of Ro, which indicated that this metabolite had been recruited into the circulation from where it was produced. OA and glycosyl oleanolate might also have been produced after the intraperitoneal administration of Ro, but the noise in the chromatogram prevented their detection. These results demonstrate that R1, IVa, and E are the metabolites and deglycosylation products of Ro, formed by the cleavage of glucose and glycolic acid at C-3 and/or C-28. It has been reported that these metabolites can be produced by intestinal microflora. R1 is degraded by intestinal microbiota and then converted into E [75]. In this study, IVa, R1, and E all reduced tumour weight in vivo. Therefore, E was thought to be the main factor leading to the anti-tumour activity after intraperitoneal administration of Ro. Based on the abovementioned results, we deem that the anti-tumour effects of Ro are based on its metabolites. Since studies of parent ginsenoside activities in vitro may not accurately depict their pharmacological effects in vivo conferred by metabolite effects, the metabolism of ginseng compound should be further investigated. In fact, Ro can also convert to IVa, R1, and E in vitro. After ginseng is steamed at 120 °C for 6 h, Ro levels sharply decrease, and the contents of IVa, R1, and E increase [76]. The improvement of biological activity after ginseng is steamed is possibly attributed to this form of composition transformation.

Angiogenesis is an essential component of tumour growth and metastasis and is a key point in the control of cancer progression. Its inhibition represents a significant new approach for improving anti-angiogenic and anti-cancer activities. Several anti-cancer drugs are specifically designed to interfere with the biochemical stimuli regulating angiogenesis. The present data proved that the three metabolites of Ro exhibit anti-angiogenic activity and inhibit the tube formation of HUVECs. Therefore, our findings suggest that R1-, IVa-, and E-mediated inhibition of B16F10 melanoma is associated with their anti-angiogenic activity. Haemolysis of the blood is an important problem associated with the bioincompatibility of materials. The haemolysis results in this experiment showed that Ro will not cause haemolysis. Thus, Ro displays excellent hemocompatibility and may serve as a promising platform for cancer treatment.

## 4. Materials and Methods

### 4.1. Reagents, Cell Lines, and Animals

Fetal bovine serum (FBS) and Dulbecco’s Modified Eagle’s Medium (DMEM) were purchased from Gibco (BRL Co. Ltd., Gaithersburg, MD, USA). MTT was purchased from Sigma (St. Louis, MO, USA). Ro, R1, IVa, and E were purchased from Chemfaces (Wuhan, China). The purities of all of the compounds were higher than 98%. B16F10 melanoma cell lines and HUVECs were purchased from the Type Culture Collection of the Chinese Academy of Sciences (Shanghai, China). The cells were cultured in DMEM containing 10% FBS (*v/v*) routinely supplemented with penicillin and streptomycin. Both B16F10 cells and HUVECs were cultured at 37 °C in a humidified atmosphere containing 5% carbon dioxide (CO_2_). Male C57BL/6J mice (6–8 weeks old) and male Sprague Dawley rats (10 weeks old) were purchased from Vital River Laboratories (License No. SCXK (Jing) 2012-0001; Beijing, China). The animals were maintained in plastic cages with a 12 h light/dark cycle and given free access to food and water. All animals were handled in strict accordance with good animal practice according to the Animal Ethics Procedures and Guidelines of the People’s Republic of China, and the study was approved by The Animal Administration and Ethics Committee of the Institute of Special Animal and Plant Sciences, Chinese Academy of Agricultural Sciences (Permit No. ISAPSAEC-2018-001).

### 4.2. Tumour Xenograft Experiment

An anti-tumour assay was conducted in a mouse xenograft model [23]. B16F10 cells were cultured in DMEM containing 10% FBS. After the collection of cells, the cells were re-suspended in normal saline to the appropriate concentrations. Next, 0.1 mL of the B16F10 cell suspension (1 × 10^6^ cells) was subcutaneously injected via the right axillary region of the C57BL/6J mice. The tumour-bearing mice were randomly divided into three groups (*n* = 6/group): model control group, positive control group, and Ro (25 mg/kg) administration group. In addition, a group of mice that did not receive tumour inoculation was used as the normal control. Normal and model mice were administered normal saline. The mice in the positive control group received CTX (20 mg/kg). The successive intraperitoneal injection of drugs into the mice from the day after tumour inoculation and the body weights were monitored every 2 days. After 15 days, all mice were sacrificed, and tumours were taken out and measured as previously described [77]. The thymus and spleen were also removed and weighed to calculate the thymus and spleen indices. The tumour inhibition rate was calculated. The blood samples were collected from the retrobulbar vessels and added into tubes containing heparin. Subsequently, plasma samples were prepared by centrifugation for 10 min at 3800 rpm and stored at −80 °C for HPLC-MS analysis.

### 4.3. Effects of Ro on B16F10 Cell Viability

In this study, Ro was dissolved in dimethyl sulfoxide (DMSO) and diluted in DMEM and then filtered using a 0.22 μm membrane. The final concentration of DMSO in cell culture medium was 0.1%, and 0.1% DMSO-treated cells were used as controls. The effect of Ro on B16F10 cell growth in vitro was performed using the MTT assay [78]. Briefly, exponentially growing B16F10 (1 × 10^4^ cells/well) cells were seeded onto 96-well plates and incubated for 24 h in complete medium. After 24 h incubation, the cells were treated with various concentrations of Ro (0, 1, 3, 10, 30, 100 μg/mL) for 24 h, and MTT (0.5%, 20 μL) was then added to each well and incubated for an additional 4 h at 37 °C. The formazan crystals were dissolved in DMSO (150 μL), and the absorbance at 490 nm was measured using a microplate reader (BioTek Epoch; BioTek, Winooski, VT, USA)

### 4.4. Determination of Ro Metabolites by HPLC-MS

Identification of the metabolites of Ro was done using the same HPLC Trap MS system as previously described [62]. Briefly, an aliquot of 60 μL plasma samples was removed from the −80 °C storage and thawed under ambient temperature and mixed vigorously with 300 μL cold methanol. The supernatant was placed in a centrifuge tube (1500 μL) after 10 min of centrifugation at 10,000 rpm. The precipitate was washed three times using 100 μL cold methanol. All of the supernatants were combined and dried under nitrogen flow at ambient temperature. The residue was dissolved using 80 μL methanol (3 min of vortexing), and the solution was diluted in 240 μL pure water. The mixture was centrifuged at 10,000 rpm for 10 min. All of the supernatant was loaded onto a flash column (25 × 2 mm id, Chromolith; Merck, Darmstadt, Germany). This flash column was connected to an analytical column (50 × 2 mm id, Chromolith; Merck) for HPLC-MS analysis. The separation was conducted using a gradient mobile phase consisting of water (A, containing 0.01% ammonium hydroxide) and acetonitrile (B). The program of the mobile phase was as follows: 10% B for the first 5 min; then a linear gradient of 10–15% B for 5–7 min, 15–40% B for 7–40 min, and 40–70% B for 40–45 min; this composition was maintained for 45–60 min and then returned to the initial condition for 5 min. The flow rate was 0.2 mL/min. The method for the Trap MS detection was the same as previously described [62].

### 4.5. Cell Viability Assay for the Ro Metabolites

The metabolites (standard substance) of Ro were dissolved in the same manner as Ro. Briefly, IVa, R1, and E were dissolved in dimethyl sulfoxide (DMSO), diluted in DMEM, and then filtered using a 0.22 μm membrane. The final concentration of DMSO in cell culture medium was 0.1%, and 0.1% DMSO-treated cells were used as controls. The effects of IVa, R1, and E on B16F10 cell growth were evaluated using the MTT assay as previously described. The cells were treated with various concentrations of IVa (0, 1, 3, 10, 30, 100 μg/mL), R1 (0, 5, 10, 20, 30, 40 μg/mL), and E (0, 2, 4, 6 μg/mL) for 24 h. After incubation, MTT (0.5%, 20 μL) was added and incubated for 4 h at 37 °C. Then the supernatants were carefully removed, and 150 μL DMSO was added to each well. After 10 min of incubation and vibration, the absorbance was read at a wavelength of 490 nm. IC50 values were calculated using Probit Analysis with SPSS 22.0 (SPSS Inc., Chicago, IL, USA).

### 4.6. Anti-Tumour Assay for the Metabolites of Ro In Vivo

A xenograft experiment was performed using the same method as described in Section 2.2. Briefly, after the mice were inoculated with tumour cells, they were randomly divided into six groups (*n* = 6/group): model control group, positive control group (CTX, 20 mg/kg), R1 (25 mg/kg), IVa (25 mg/kg), and E (5 mg/kg) administration groups. In addition, a normal control group of mice did not receive tumour inoculation. Normal and model mice were administered normal saline. Drugs were intraperitoneally injected into the mice for 15 consecutive days. Tumour volumes were measured every 2 days, and tumour growth curves were plotted. At the end of the experiment, the mice were sacrificed, and the tumour weight and tumour growth inhibition rates were calculated.

### 4.7. Tube Formation Assay

The tube formation assay was performed as previously described with some modifications [79]. Matrigel (BD Biosciences, San Jose, CA, USA) was thawed overnight at 4 °C. Next, 50 μL Matrigel was coated on each well of the pre-chilled 96-well plates and then incubated at 37 °C for 1 h. HUVECs (5 × 10^4^ cells/well) were seeded on the Matrigel and treated with various concentrations of Ro (0, 1, 3, 10, 30, 100 μg/mL), R1 (0, 5, 10, 20, 30, 40 μg/mL), IVa (0, 1, 3, 10, 30, 100 μg/mL), and E (0, 2, 4, 6, 8, 10 μg/mL). After incubation for 5 h, the formation of HUVEC tubular structures was captured using a microscope (Nikon eclipse Ti; Nikon, Tokyo, Japan). The tube length was quantified using Image-Pro Plus 6.0 software.

### 4.8. Haemolysis Test

To evaluate the haemocompatibility of Ro, the haemolysis test was performed as previously described [80]. Arterial blood was obtained from healthy Sprague Dawley rats. Then erythrocytes were separated from the plasma and lymphocytes by centrifugation (3000 rpm, 5 min) at 4 °C, washed three times with normal saline, and suspended in normal physiological saline at a haematocrit of 2%. Erythrocytes were used immediately after isolation. Next, 2.5 mL erythrocyte suspension and 2.5 mL medicated saline solution were added to a 10 mL centrifuge tube. The final concentrations of Ro were 1, 3, 10, 30, and 100 μg/mL. The negative and positive controls were normal physiological saline and 0.2% Triton X-100, respectively. The samples were incubated for a specific time period at 37 °C for 60 min. Next, the tube was centrifuged at 3000 rpm for 5 min. Finally, the optical density (OD) was obtained at a wavelength of 545 nm. Three parallel samples were laid in each group. The mean value of the three samples was obtained as the group OD value. The HR was expressed as the percentage and calculated according to the equation:

HR = (ODt − ODn)/(ODp − ODn) × 100%,(1)

where ODt indicates the OD value of the tested group, and ODn and ODp are the OD values of the negative and positive control groups, respectively. A haemolysis rate less than 5% is regarded as a nontoxic effect level.

### 4.9. Statistical Analyses

Statistical analyses were performed with SPSS 22.0 and GraphPad Prism 6.0 (GraphPad Software, La Jolla, CA, USA). All data were expressed as the mean ± standard deviation (SD). Data were analysed by ANOVA with Tukey’s multiple comparison test and Dunnet’s multiple comparison test. Differences were considered significant if *p* < 0.05 and extremely significant if *p* < 0.01.

## 5. Conclusions

The results of this study clearly demonstrate that Ro inhibits tumour growth in B16F10 tumour-bearing mice but has no effects on B16F10 cell growth in vitro. IVa, R1, and E, metabolites of Ro, had anti-tumour effects both in vitro and in vivo and also inhibited angiogenesis to some extent. Our findings provide substantial evidence that Ro might be beneficial for the therapeutic management of melanoma. These findings indicate that the metabolites of Ro may underlie its pharmacological anti-cancer mechanisms.

## Figures and Tables

**Table 1:** Effects of Ro on body weight and organ indices in B16F10 tumour-bearing mice.

Groups	Dosage (mg/kg)	Weight (g)		Organ Indices (×100, mg/g)	
		Before	After	Thymus	Spleen
Normal	-	23.11 ± 0.67	24.27 ± 0.99	1.97 ± 0.14	3.46 ± 0.33
Model	-	22.27 ± 0.71	24.89 ± 1.26	1.23 ± 0.27 ^##^	4.74 ± 1.21 ^#^
CTX	20	22.29 ± 0.85	22.27 ± 1.02	0.56 ± 0.09 **	2.98 ± 0.86 *
Ro	25	22.91 ± 1.12	24.73 ± 1.43	1.67 ± 0.25 *	3.54 ± 1.03

^#^*p* < 0.05, ^##^ *p* < 0.01 vs. normal group; * *p* < 0.05, ** *p* < 0.01 vs. model group.

**Table 2:** Haemolysis rate of Ro at different concentrations.

Ro (μg/mL)	Haemolysis Rate (%)
1	0.15 ± 0.13
3	0.31 ± 0.13
10	0.23 ± 0.23
30	0.15 ± 0.13
100	0.31 ± 0.35

## Supplementary Materials

The following are available online, Figure S1: The skeleton structures of ginsenosides. (A) PPD-type; (B) OA-type. R1, R2, and R3 are the sites of sugar attachment on the skeleton of ginsenosides. Sugar moieties are listed in Table S1. Table S1: Summary of ginsenoside structures.
