# Supplementary material for: Inhibitory Effects of Ginsenoside Ro on the Growth of B16F10 Melanoma via Its Metabolites
Source: Molecules. 2019 Aug 17;24(16):2985. doi: 10.3390/molecules24162985 (PMC6721120; doi:10.3390/molecules24162985)
Supplement: Supplementary file 1 [file molecules-24-02985-s001.pdf]

molecules

MDPI

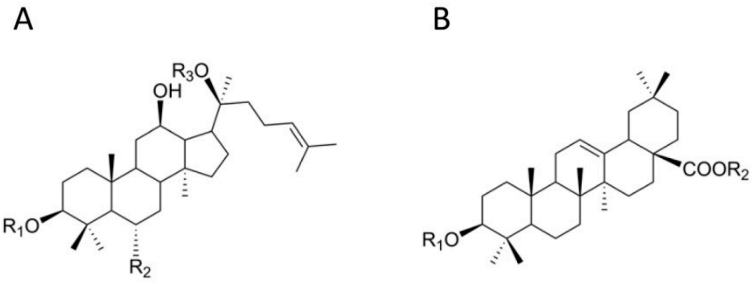

Figure S1. The skeleton structures of ginsenosides. (A) PPD-type; (B) OA-type. R1, R2 and R3 are the sites of sugar attachment on the skeleton of ginsenosides. Sugar moieties are listed in Table S1.

Table S1. Summary of ginsenoside structures

Group	Ginsenoside	Carbohydrate Moieties		
		R1	R2	R3
PPD-type	Rb1	Glc-Glc	H	Glc-Glc
	CK	H	H	Glc
OA-type	Ro	GlcUA-Glc	Glc	-
	R1	GlcUA-Glc	H	-
	IVa	GlcUA	Glc	-
	E	GlcUA	H	-

Glc: β-D-glucopyranosyl; GlcUA: β-D-gulcuronic acid
